# Editorial: Molecular mechanisms and pathways in cerebellar function

**DOI:** 10.3389/fnmol.2023.1258215

**Published:** 2023-07-28

**Authors:** Catarina Osório, Alanna J. Watt, Lilian Kisiswa

**Affiliations:** ^1^Erasmus Medical Center, Rotterdam, Netherlands; ^2^Department of Biology, McGill University, Montreal, QC, Canada; ^3^Department of Biomedicine, Aarhus University, Aarhus, Denmark; ^4^Danish Research Institute of Translational Neuroscience (DANDRITE)–Nordic EMBL Partnership for Molecular Medicine, Aarhus University, Aarhus, Denmark; ^5^The Danish National Research Foundation Center, PROMEMO, Aarhus University, Aarhus, Denmark

**Keywords:** neurodevelopment, signaling pathways, plasticity, cerebellar disorders, cerebellar therapies

The cerebellum contains the majority of the neurons found in the central nervous system. This neuronal richness poses a challenge for the correct wiring of the cerebellar neurocircuits that are a prerequisite for a properly functioning cerebellum. These circuits underlie a wide range of functions such as motor learning, balance, and coordination, as well as cognitive and emotional processes. Strikingly, the human cerebellum has a prolonged developmental period, which increases the risk for both genetic and environmental disruptions, and consequently neurological disorders. Hence, a deeper knowledge on the fundamental molecular and cellular processes governing cerebellar development is imperative, and may eventually provide a springboard for novel treatments. With this Research Topic we provide a platform to bring together the latest findings focused on the molecular and cellular mechanisms underlying cerebellar development, function and disease.

The molecular mechanisms driving cerebellar developmental programs are not fully understood. However, studies from several groups are now elucidating these mechanisms and pathways. Focusing on the granule cells (GCs), Kim et al. summarize the GCs-related molecules involved in cerebellar development. GCs constitute more than 50% of the brain's neurons, hence their development influence not only the size and foliation of the cerebellum but also the development of other neurons. Kim et al. proposed several research directions to further investigate how GCs regulate the assembly of the cerebellar network. Importantly, combining neurodevelopmental studies with behavioral assays allows for a better understanding on the impact specific molecules have in the maturation and function of the cerebellum. For instance, the absence of the neurotrophin receptor p75 (p75^NTR^) from granule cell precursors (GCPs) increases their rate of proliferation. Consequently, mice with an excess of GCs performed poorer in the eyeblink conditioning, a cerebellar-dependent motor learning task. Building on their previous work, Zanin et al. tested the same p75^NTR^ model for non-motor behaviors. These animals displayed high levels of anxiety, lack of preference on social novelty and social disengagement. Overall, deletion of p75^NTR^ from GCPs during development was sufficient to perturb social behavior. The involvement of p75^NTR^ suggests that the neurotrophins play an important role in cerebellar development. This is further supported in a systematic review by Boxy et al. that detailed how each trophic factor participate in the survival, migration, differentiation, synaptogenesis, and maintenance of the cerebellum. They provide examples on how disruption of their signaling pathways contribute for neurodevelopment disorders with a cerebellar component. These articles highlight the need to understand how disruptions in signaling pathways lead to brain abnormalities so better therapeutic strategies can be developed.

A mechanistic study presented by Okuno et al. focused on the assembly of cerebellar neuronal networks. The climbing fiber to Purkinje cell (PC) synapse is a well-established model for synaptogenesis: it develops from early multi-innervating climbing fibers to the selective strengthening of one climbing fiber input, and finally to synapse elimination of extra climbing fibers. Perturbations in any of these steps can have dramatic consequences on cerebellar connectivity. Okuno et al. report the receptor-type tyrosine-protein phosphatase delta (PTPδ) as a novel presynaptic organizer in the cerebellum. Using a combination of mouse genetics, molecular and systems neurosciences approaches, they showed that PTPδ is required for both motor function as well as during development for climbing fiber translocation, synaptic transmission and maintenance predominantly in the anterior cerebellum. It is important to recognize that the cerebellum is not a homogeneous structure and many studies have revealed molecular, cellular, physiological and functional regional differences. However, the mechanisms underlying these emerging differences during development are still unclear. In this topic, Dorigo et al. used zebrafish as a developmental model to shed light on the origins of cerebellar functional domains. Pharmacological as well as optogenetic silencing of PC activity disrupted the development of functional regionalization of the PC layer. The authors propose an activity-driven maturation for cerebellar connectivity in which PC physiological response develops alongside their functional regionalization.

Given the importance of proper developmental programs in establishing cerebellar neuronal connections, alterations in the molecular composition of neuronal circuits can result in cerebellar diseases. A stellar example of disorders caused by impairment in neurocircuits are the spinocerebellar ataxias (SCAs) that comprises of a group of degenerative diseases originated by a wide range of mutations. Kapfhammer and Shimobayashi summarized the evidence that mutation in Protein kinase C gamma (PKCγ) results in a constitutive active protein and this alters PC function and signaling, which in turn impairs cerebellar circuits causing SCA. Disruptions in cerebellar circuitry was further demonstrated in Piasecki et al. where they showed that ataxin-3 containing 150 CAG repeats leads to impaired ataxin-3 interactome. Loss of ataxin-3/Camk2 interaction impeded the transport of mitochondria to axons. Insufficient axonal mitochondrial transport reduces local energy supply leading to axonal degeneration. Additionally, Gorski et al. reported that mutation in cystatin B (*CSTB*) gene leads to mitochondria dysfunction that perturbs synaptic function resulting in ataxia. Impairment in mitochondrial function is extended to other forms of ataxia. Chen et al. identified a patient carrying a novel homozygous senataxin (*SETX*) mutation that causes autosomal recessive inherited ataxia with oculomotor apraxia type 2. Non-functioning *SETX* gene causes protein aggregation and accumulation of defective mitochondria, which subsequently results in neurite degeneration that alters the cerebellar circuit. Data on the molecular signatures that drive impairment in cerebellar circuits described here may eventually give rise to novel therapeutic interventions in SCA patients.

Indeed, Ding et al. reviewed the basis and prospect of therapies for SCAs focusing on the potential use of RNA interference (RNAi) and extracellular vesicles (EVs). They proposed a novel therapeutic strategy that combines RNAi and EVs for the treatment of SCAs. Although RNAi treatment has been explored in patients and mouse models of SCAs with some success, RNAi poor stability *in vivo* and toxicity caused by excess of RNAi makes the use of RNAi technology alone insufficient for treating SCA patients. Therefore, packing EVs with RNAi may be a good strategy to increase RNAi stability and reduce toxicity *in vivo* resulting in an increased potential of gene therapy for the treatment of these diseases. Other cerebellar-related disorders such as Down Syndrome (DS) also exhibit mitochondrial dysfunction and impairment in neuronal circuits. DS caused by overexpression of dual specific tyrosine-phosphorylation-regulated kinase 1A show alteration in the cerebellar proteome indicating mitochondrial dysfunction that could be rescued by green tea extract containing epigallocatechin-3-gallate as evidenced in Ortega et al.. Taken together, these articles elucidate molecular mechanisms of mitochondrial dysfunction altering cerebellar network associated with cerebellar malfunction. This knowledge may aid the development of therapies that can halt disease progression and eventually provide relief to patients.

Once the cerebellar network is established, it requires maintenance across the lifespan of an animal. Butler et al. demonstrated that subjecting the developing cerebellum to hemorrhagic injury, leads to reduced number of cerebellar neurons but this did not alter the behaviors of the animals suggesting compensatory robustness in the network. Furthermore, the dynamic nature of neurocircuits suggests that there are mechanisms in place to ensure that circuit function is maintained in adulthood. Although, factors required for maintenance of healthy cerebellar circuits are partially known, van't Sant et al. provided additional evidence that dietary restriction promotes expression of genes that contributes to maintenance of these circuits.

Overall, a proper functioning cerebellum requires correct wiring of circuits that are built by a combination of molecular neurodevelopmental programs and mechanisms that maintain these circuits. This Research Topic provides a glimpse of the molecular mechanisms responsible for the development of cerebellar circuits and their preservation in adulthood. These studies emphasize the need for further studies to understand the formation and maintenance of cerebellar wiring and elucidate what goes wrong in cerebellar-related disorders. This knowledge will provide a foundation for development of therapeutic interventions for cerebellar diseases.

## Author contributions

All authors listed have made a substantial, direct, and intellectual contribution to the work and approved it for publication.

